# Comprehensive analysis of the catalytic and structural properties of a mu-class glutathione s-transferase from *Fasciola gigantica*

**DOI:** 10.1038/s41598-017-17678-3

**Published:** 2017-12-13

**Authors:** Jupitara Kalita, Rohit Shukla, Harish Shukla, Kundlik Gadhave, Rajanish Giri, Timir Tripathi

**Affiliations:** 1 Molecular and Structural Biophysics Laboratory, Department of Biochemistry, North-Eastern Hill University, Shillong, 793022 India; 2 School of Basic Sciences, Indian Institute of Technology Mandi, Kamand, Himachal Pradesh 175005 India

## Abstract

Glutathione S‒transferases (GSTs) play an important role in the detoxification of xenobiotics. They catalyze the nucleophilic addition of glutathione (GSH) to nonpolar compounds, rendering the products water-soluble. In the present study, we investigated the catalytic and structural properties of a mu-class GST from *Fasciola gigantica* (FgGST1). The purified recombinant FgGST1 formed a homodimer composed of 25 kDa subunit. Kinetic analysis revealed that FgGST1 displays broad substrate specificity and shows high GSH conjugation activity toward 1-chloro-2,4-dinitrobenzene, 4-nitroquinoline-1-oxide, and trans-4-phenyl-3-butene-2-one and peroxidase activity towards trans-2-nonenal and hexa-2,4-dienal. The FgGST1 was highly sensitive to inhibition by cibacron blue. The cofactor (GSH) and inhibitor (cibacron blue) were docked, and binding sites were identified. The molecular dynamics studies and principal component analysis indicated the stability of the systems and the collective motions, respectively. Unfolding studies suggest that FgGST1 is a highly cooperative molecule because, during GdnHCl-induced denaturation, a simultaneous unfolding of the protein without stabilization of any partially folded intermediate is observed. The protein is stabilized with a conformational free energy of about 10 ± 0.3 kcal mol^−1^. Additionally, the presence of conserved Pro-53 and structural motifs such as N-capping box and hydrophobic staple, further aided in the stability and proper folding of FgGST1.

## Introduction

Fascioliasis, a neglected tropical disease, is caused by the food-borne trematodes *Fasciola hepatica* and *Fasciola gigantica*. They are one of the most important pathogen of domestic livestock with global distribution. These parasites infect mammals through ingestion of aquatic plants or contaminated water having encysted metacercariae. The life cycle of these parasites requires two hosts: lymnaeid snails as intermediate host and mammals as definitive host. *F*. *hepatica* causes fascioliasis in colder climates while *F*. *gigantica* infection is confined to the tropical regions of Africa, the Middle East, and Asia; its highest prevalence has been reported in Bolivia^1–4^. Unlike other helminth infections, fascioliasis does not respond to treatment with albendazole or praziquantel. The WHO recommends triclabendazole for the treatment of fascioliasis^5^. However, recent studies have suggested that these parasites have gained resistance to triclabendazole in several countries^6–8^.

Glutathione transferases (GSTs; EC 2.5.1.18) are widely distributed in nature and present in prokaryotes to most complex eukaryotes^9^. GSTs catalyze the glutathionylation by adding glutathione (GSH) to an electrophilic center of their substrates^10^. They play a key role in the Phase II of cellular detoxification process^11^. GSTs are involved in the removal of potentially toxic chemicals such as xenobiotics, drugs, chemical carcinogens, and environmental pollutants^12^. They can also reduce lipid peroxidation products formed by free radical attack on water-soluble compounds^13^. GSTs are classified into four major groups based on substrate specificity: cytosolic GSTs, kappa-class GSTs (mitochondrial), membrane-associated proteins in eicosanoid and glutathione metabolism (MAPEG, microsomal), and bacterial fosfomycin-resistant proteins^14–17^. The cytosolic GSTs are more abundant and can be further divided into several classes like mu, alpha, pi, theta, sigma, zeta, omega, nu, lambda, phi, tau, delta, epsilon, iota, chi, and rho^10,12,18–25^. Structurally, most of the GSTs are dimeric that can be either homodimer or heterodimer. Each monomer consists of two distinct N-terminal and C-terminal domains. The N-terminal domain is similar to the thioredoxin fold that consists of four β-sheets with three flanking α-helices^12,22^, where the GSH molecule binds (G-site). In contrast, the electrophilic compounds bind to all α-helical C-terminal domain (H-site)^26^. Most of the structural variations are present in the C-terminal domain, which enables the enzyme to bind a wide range of electrophilic compounds^19,20^.

Helminth parasites express GSTs in response to drug treatment^27^. Hence, GSTs are necessary for the survival of these parasites as they face several challenges such as the host’s diet, components of the immune response of the host and from antihelminthics administered to kill the parasite. Many GSTs from helminths such as *Schistosoma*, *Ascaris*, and *Onchocerca* species have been characterized, and crystal structures are available for several of them^28–31^. The main cytosolic GST classes identified in helminth parasites are mu, pi, and sigma, along with some alpha and omega class GSTs^27,32,33^ A characteristic feature of mammalian mu-class GSTs is the presence of a mu-loop between β2 strand and α2 helix. This mu-loop is not present in helminth mu-GSTs like Fh47GST and SjGST. The absence of this loop makes G-site more accessible in these parasitic GSTs. Helminth GSTs are an important target for chemotherapeutic and vaccine development. A cytosolic GST from *S*. *haematobium* (Sh28GST) has been reported as an important vaccine candidate and has completed phase I of clinical trials^27^. In *F*. *hepatica*, both native and adjuvant GSTs have been reported to provide significant protection against liver fluke infection in sheep^34^. In the present study, we systematically characterized the structural and functional properties of a mu-class GST from *F*. *gigantica*.

## Results and Discussion

### Sequence analysis and phylogenetic relationship

**Figure 1:**
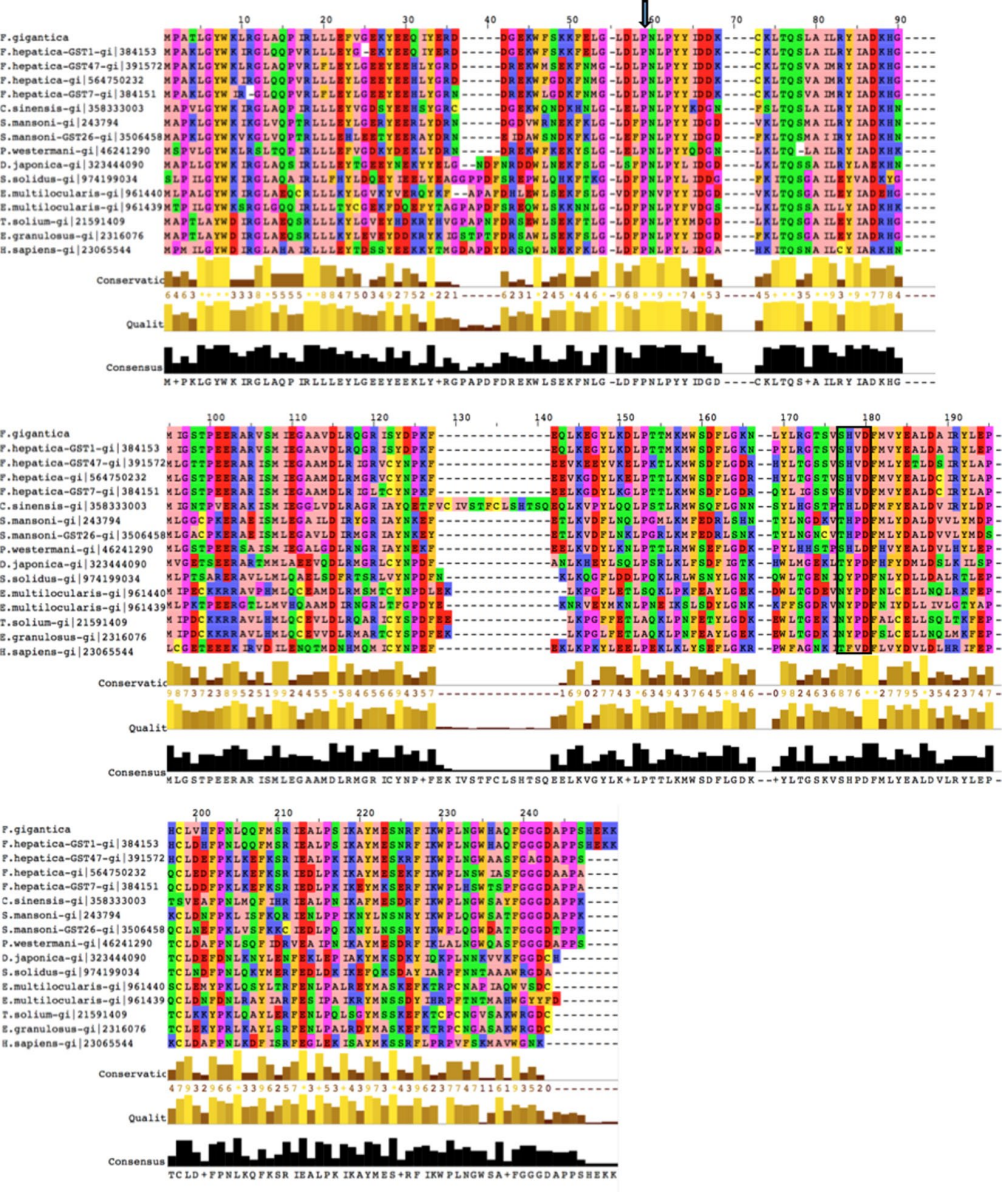
Sequence alignment of various mu-class GSTs. Alignment of FgGST1 amino acid sequence with other mu-class GSTs retrieved from the NCBI database. The alignment was generated by ClustalW algorithm. *F*. *hepatica*, *P*. *westermani*, *C*. *sinensis*, *S*.*mansoni*, *P*. *westermani*, *D*. *japonica*, *S*. *solidus*, *E*. *multilocularis*,*T*. *solium*, *E*. *granulosus* and *H*. *sapiens* were included in the alignment. Arrow represents the conserved Pro-53, while the box represents residues of the N-capping box and hydrophobic staple.

Multiple sequence alignment showed that FgGST1 has the highest sequence identity with FhGST (96.36%). FgGST1 showed 76.39%, 75%, 72.69%, 68.98%, 64.78%, 60.19%, 55.09%, 48.61%, 46.79%, 44.19%, 44.50%, 42.86%, 42.40%, and 46.3% identity with FhGST47, FhGST-mu, FhGST7, *P*. *westermani*, *C*. *sinensis*, *S*. *mansoni*, *P*. *westermani*, *D*. *japonica*, *S*. *solidus*, *E*. *multilocularis*, *E*. *multilocularis*-mu1, *T*. *solium*, *E*. *granulosus*, and *H*. *sapiens* GST, respectively (Fig. 1). The phylogenetic tree showed that FgGST1 shared the evolutionary clade with FhGST1-mu and is very distinct from the human GSTs (Supplementary Figure S1).

The amino acid sequence alignment showed that the conserved Pro-53 is also present in FgGST1. Crystal data from several GSTs indicates that this Pro-53 adopts the cis-configuration. Pro-53 is located in a β-turn that lines the base of the G-site and is important for the proper folding and maintenance of conformation of the G-site. The structural motifs, denoted as N-capping box and hydrophobic staple that are crucial for the folding of GSTs, are conserved and present in FgGST1. Asp-140 residue, which is a part of the N-terminal box, is thought to be involved in the stability and structural maintenance of GSTs^35–37^. The sequence alignment supports the idea that these residues were conserved during evolution because of their involvement in the folding and stability of cytosolic GSTs.

### Purification and structural characterization of FgGST1

**Figure 2:**
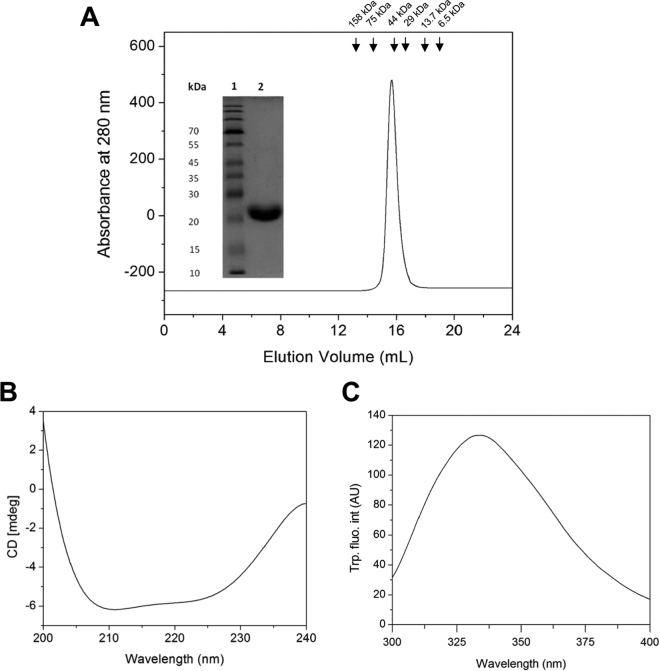
Purification and structural features of recombinant FgGST1. (**A**) SEC profile. Inset shows the SDS-PAGE profile of purified protein. Lanes 1 and 2 represent molecular weight marker and purified FgGST1 respectively. The column was calibrated with standard molecular weight markers: Aldolase (158 kDa), conalbumin (75 kDa), ovalbumin (44 kDa), ribonuclease A (13.7 kDa) and aprotinin (6.5 kDa). (**B**) Far-UV CD spectrum. (**C**) Tryptophan fluorescence spectrum.

FgGST1 was over-expressed and purified as described in the experimental section. The yield of purified recombinant FgGST1 protein was approximately 50 mg/L culture. The molecular mass of the purified protein was determined by SDS-PAGE that showed a 25 kDa protein band (Fig. 2A inset). The quaternary structure of FgGST1 was determined by using SEC. The FgGST1 eluted at an elution volume of 15.4 mL that corresponds to about 50 kDa when compared with the molecular weight markers. This suggests that under non-denaturating conditions, the protein exists in a dimeric state in the solution (Fig. 2A).

The secondary structure of FgGST1 was predicted by using far-UV CD. It is established that polypeptides and proteins show a characteristic far-UV CD spectra for α-helical and β-sheet proteins, with α-helical proteins having two minima at 222 and 208 nm and β-sheet proteins having a single minima at 216 nm. A far-UV CD spectrum of FgGST1 demonstrates the presence of both α-helices and β-sheets in the secondary structure (Fig. 2B). The tertiary structure of FgGST1 was determined by using intrinsic Trp fluorescence. According to the amino acid sequence, FgGST1 has four Trp residues at positions 8, 133, 201 and 206. Native FgGST1 showed the emission maximum at about 334 nm (Fig. 2C). The buried Trp residues in a folded protein show fluorescence emission maximum at 330–335 nm, suggesting that the Trp residues in FgGST1 are significantly buried in the protein core.

### Effects of pH and temperature on enzymatic activity

**Figure 3:**
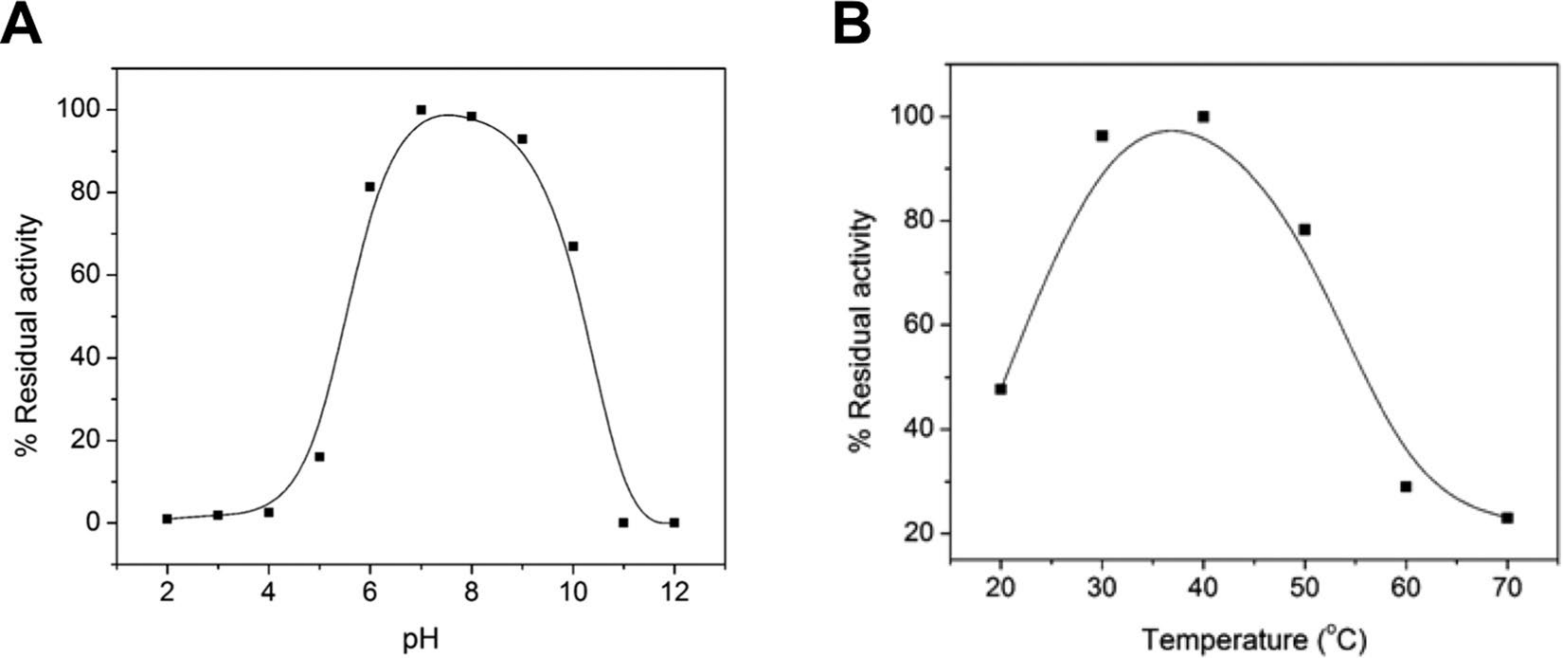
Effect of pH and temperature on the enzymatic activity of FgGST1. (**A**) Effect of pH on catalytic activity of FgGST1. (**B**) Effect of temperature on catalytic activity of FgGST1. Experiments were repeated thrice and ±SD was taken.

The pH optimum of FgGST1 with CDNB as substrate was found to be 7.5. At the pH value below 6.0 and above 9.0, the activity decreased substantially (Fig. 3A). Temperature dependent studies revealed maximum FgGST1 activity at 40 °C while the activity was reduced to approximately 50% at 20 °C. In addition, the activity decreased significantly at high temperatures, i.e., the activity reduced to 25% at 60 °C and 20% at 70 °C (Fig. 3B).

### Substrate specificity of FgGST1

**Figure 4:**
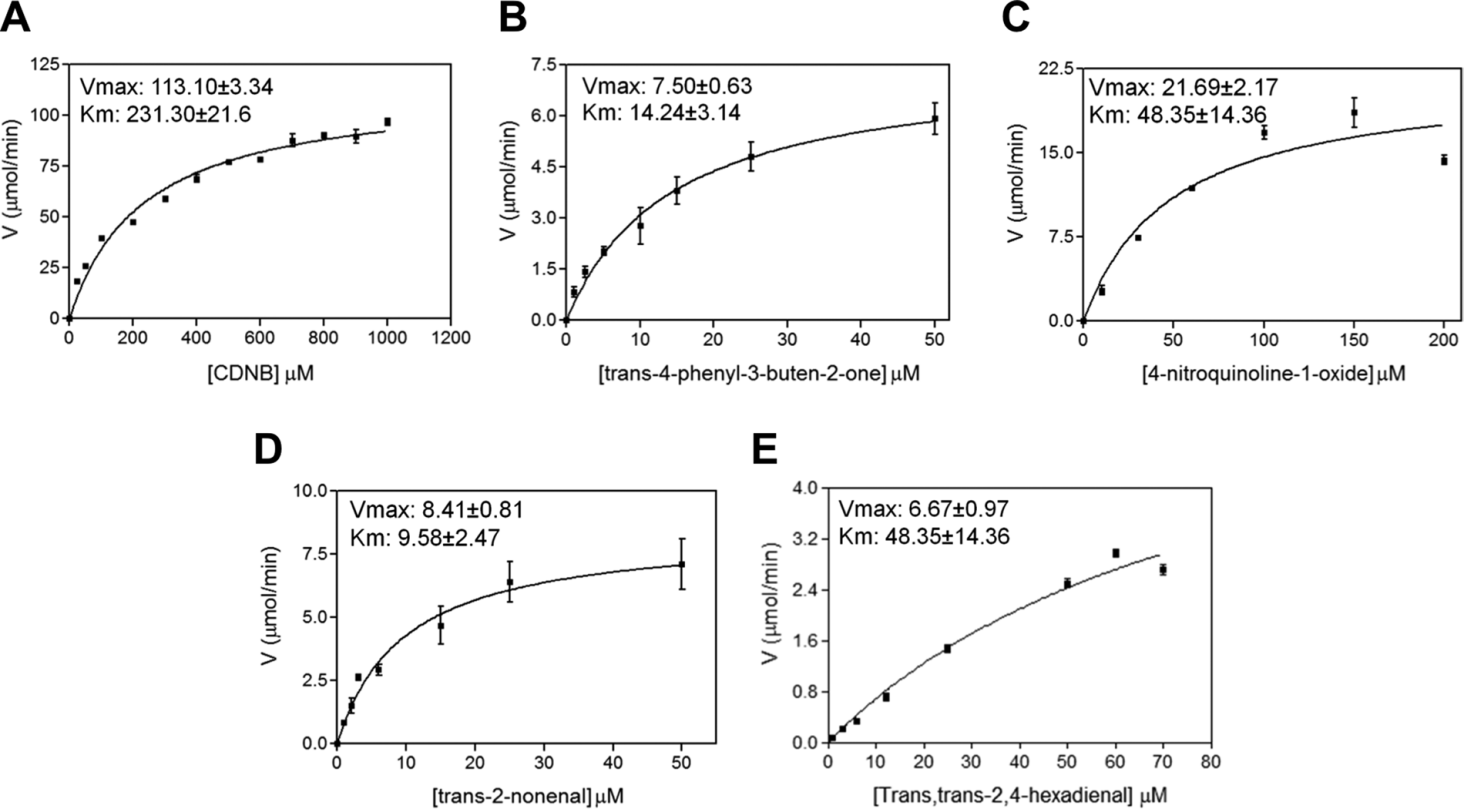
Michaelis-Menton plots. Catalytic activity of FgGST1 with increasing concentration of the substrates-(**A**) CDNB. (**B**) Trans-4-phenyl-3-buten-2-one. (**C**) 4-Nitroquinoline-1-oxide. (**D**) Trans-2-nonenal. (**E**) Trans-2,4-hexadienal. The steady state kinetic parameters were determined from the graphs.

Several other substrates were tested for activity with FgGST1, apart from CDNB (Table 1). FgGST1 was found to be active against trans-2-nonenal, hexa-2,4-dienal, trans-4-phenyl-3-butene-2-one and 4-nitroquinolone-1-oxide. The steady-state kinetics of FgGST1 with these four compounds and CDNB are shown in Fig. 4 and Table 2. The comparative account of the specific activity of FgGST1 with respect to other mu-class GSTs is shown in Table 3. FgGST1 showed significant activity with pi- and mu- class specific substrate-4-nitroquinoline-1-oxide, whereas it was moderately active with trans-4-phenyl-3-butene-2-one. FgGST1 was also found to be active against trans-2-nonenal and hexa-2,4-dienal, suggesting its role in the removal of lipid peroxidation products. Interestingly, it shows no activity against 1,2-dichloro-4-nitrobenzene (DCNB), which is marker substrate of mu-class GSTs in mammals, and with trans-stilbene oxide and ethacrynic acid, which are substrates of human mu-class GSTs^38,39^.

**Table 1:** Assay conditions and specific activity of FgGST1 with various substrates.

Substrate	[Substrate] (mM)	[GSH] (mM)	Wavelength (nm)	Extinction coefficient (ε) (mM^−1^cm^−1^)	Specific activity (μM^−1^mg^−1^min^−1^)
1-Chloro-2,4-dinitrobenzene	1.0	3.0	340	9.6	68.54 ± 2.02
1,2-dichloro-4-nitrobenzene	1.0	5.0	345	8.5	ND
trans-2-nonenal	0.025	1.0	225	−19.2	5.10 ± 0.49
Trans-4-phenyl-3-buten-2-one	0.05	0.25	290	−24.8	4.54 ± 0.41
Ethacrynic acid	0.2	0.25	270	5.0	ND
4-Nitroquinoline-1-oxide	0.25	1.0	350	7.2	13.14 ± 1.3
Trans,trans-2,4-Hexadienal	0.05	2.5	280	−34.2	4.04 ± 0.59
Bromosulfophthalein	0.03	5	330	4.5	ND

**Table 2:** Kinetic parameters.

Substrate	*V*max (μM)	*K*m (μM)	*K*cat (min^−1^)	*k*cat/*K*m (μΜ^−1^min^−1^)
1-Chloro-2,4-dinitrobenzene	113.10 ± 3.34	231.30 ± 21.60	3526.26 ± 106.07	15.24 ± 4.91
Trans-2-nonenal	8.41 ± 0.81	9.57 ± 2.46	262.51 ± 9.78	27.42 ± 3.96
4-Nitroquinoline-1-oxide	21.69 ± 2.17	48.35 ± 14.36	676.88 ± 67.46	14.00 ± 4.70
Trans-4-phenyl-3-buten-2-one	7.50 ± 0.63	14.24 ± 3.14	233.93 ± 21.27	16.43 ± 6.77
Trans,trans-2,4-Hexadienal	6.67 ± 0.97	87.48 ± 50.52	208.09 ± 30.33	2.38 ± 1.47

Enzymatic activities were measured at various concentrations of substrates. Kinetic constant are based on three independent experiments for each measurement.

**Table 3:** Comparison of specific activities. The specific activity of GSTs from different flukes with various substrates has been provided.

	Specific activity (μΜ^−1^mg^−1^min^−1^)					
	1-Chloro-2,4-dinitrobenzene	trans-2-nonenal	4-Nitroquinoline-1-oxide	Trans-4-phenyl-3-buten-2-one	Trans,trans-2,4-Hexadienal	Reference
FgGST1	68.54 ± 2.02	5.10 ± 0.49	13.15 ± 1.31	4.54 ± 0.41	4.04 ± 0.59	Current work
rFhGST47	21.00 ± 3.30	0.52 ± 0.07	NA	0.43 ± 0.1	NA	77
rcs26GST	9.17 ± 1.69	0.12 ± 0.07	NA	0.49 ± 0.11	0.18 ± 0.03	48
Sm28GST	7.27 ± 0.22	0.45 ± 0.01	NA	0.02 ± 0.00	NA	78
Sj26GST	5.09 ± 0.15	0.87 ± 0.04	NA	0.61 ± 0.04	NA	78
Pw26GST	325 ± 46	ND	NA	40 ± 13	ND	79

The GSTs include: rFhGST- recombinant *F*. *hepatica* GST, rcs26GST-recombinant *C*. *sinensis* GST, Sm28GST-*S*.*mansoni*GST, Sj26GST- *S*. *japonicum* GST, Pw26GST –*P*. *westermani* GST.

NA: not assayed.

ND: not detected.

### Inhibition studies with cibacron blue

**Figure 5:**
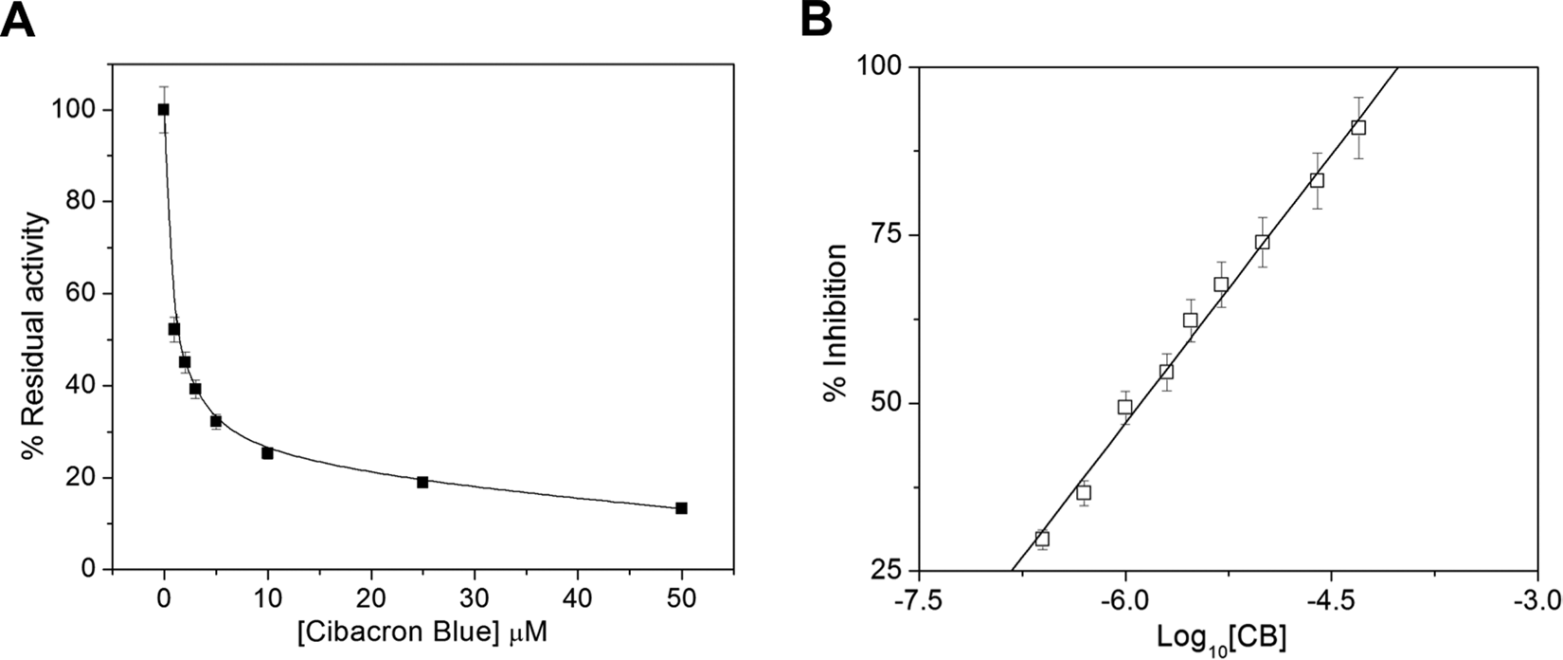
Inhibition of FgGST1 activity by cibacron blue. (**A**) Plot of percent residual activity *vs* CB concentration. (**B**) Plot of percent inhibition of FgGST1 *vs* log of CB concentrations to determine the IC_50_.

The dye cibacron blue (CB) and bromosulfophthalein are known inhibitors of mu-class GSTs. They were tested for its ability to inhibit the CDNB-conjugating activity of FgGST1. CB exhibited a concentration-dependent inhibition profile in the concentration range of 1 to 50 μM with an IC_50_ value of ~1.35 μM (Fig. 5). No significant inhibition was observed in case of bromosulfophthalein.

### Homology modeling and structure validation of FgGST1

**Figure 6:**
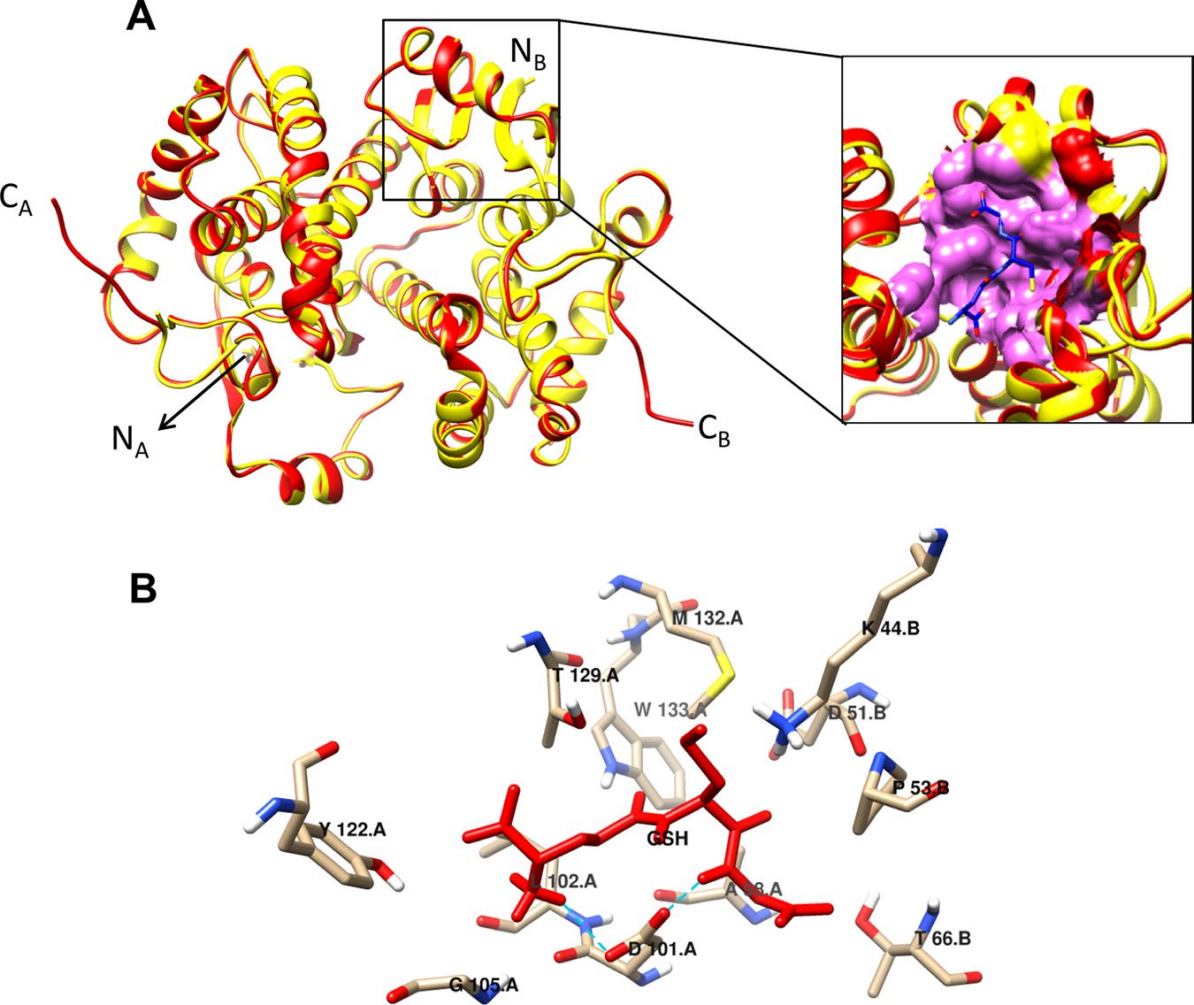
Structural features of FgGST1. (**A**) Structural alignment of FgGST1 (red) with FhGST (yellow) generates 0.152 Å RMSD for 216 atom pairs. Low RMSD value indicates structural similarity. Inset shows the docking of GSH in the cavity of FgGST1. Blue color stick indicates GSH. (**B**) Interaction of GSH with FgGST1. Dotted lines represents the H-bonds. GSH is shown in red color stick.

*In silico* secondary structure prediction of FgGST1 was done by PSI-PRED. It predicted a structure with seven α-helices and five β-sheets, which is a characteristic feature of GST structure (Supplementary Figure S2). The SOPMA server showed that α-helices, extended strands, β-turns and random coils are 50.45%, 9.91%,10.36% and 29.28% respectively for FgTGR sequence. Due to unavailability of X-ray/NMR structure of FgGST1, we modeled the 3D structure of FgGST1 using FhGST (PDBID: 2FHE, X-ray, 2.3 Å) as a template. Pair-wise alignment with FgGST1 and FhGST showed 204 identical residues (91.9%) out of 222 residues (Supplementary Figure S3), suggesting that FhGST can be considered as an ideal template for homology modeling. The homodimeric structure of FgGST1 was modeled by using Modeller9.16. The model of FgGST1 was validated by RMSD, Ramachandran plot, Z-score, and energy plot. The predicted FgGST1 model was superimposed with FhGST structure that showed an excellent RMSD value of 0.152 Å for 216 atom pairs (Fig. 6A). Then Ramachandran plot was calculated to determine the phi and psi angles. The Ramachandran plot showed 93.7% residues in the most favored region and 4.5% in the additional allowed region (Supplementary Figure S4). The ProSA software was used to calculate the Z-score and energy plot of the model. The Z-score for the template and predicted model was found to be −8.47 and −7.83, respectively (Supplementary Figure S5). This suggested that the predicted model is in good agreement with the FhGST template. The energy plot showed that all the residues lie in the negative window (Supplementary Figure S6). All the parameters suggested that predicted model was good and can be further used for docking with GSH and the inhibitor CB.

### Molecular docking with GSH

The structure of FhGST (PDB ID: 2FHE) was observed as a GSH bound complex. So we superimposed our predicted model with FhGST and selected the G-site residues to prepare the grid (Fig. 6A). GSH was docked with FgGST1 for exploring the binding pattern and interacting residues. The top pose with lowest binding energy was selected from docking. Best pose that formed the low energy complex with FgGST1 showed a binding energy of −5.7 Kcal.mol^−1^. GSH binds into the defined cavity. It interacts with residues of both A and B chains. GSH was stabilized by two hydrogen bonds with Asp101 of chain-A. It was also found to interact with Leu102, Tyr122, Thr129, Met132, and Trp133 residues of chain-A by hydrophobic interactions. GSH was stabilized by forming hydrophobic interactions with Lys44, Asp51, Pro53, and Thr66of chain-B (Fig. 6B). It is well reported that both monomers of the dimeric GSTs are involved in GSH binding; thus, our docking is in agreement with the previously reported structures^40^.

### Molecular docking with CB

**Figure 7:**
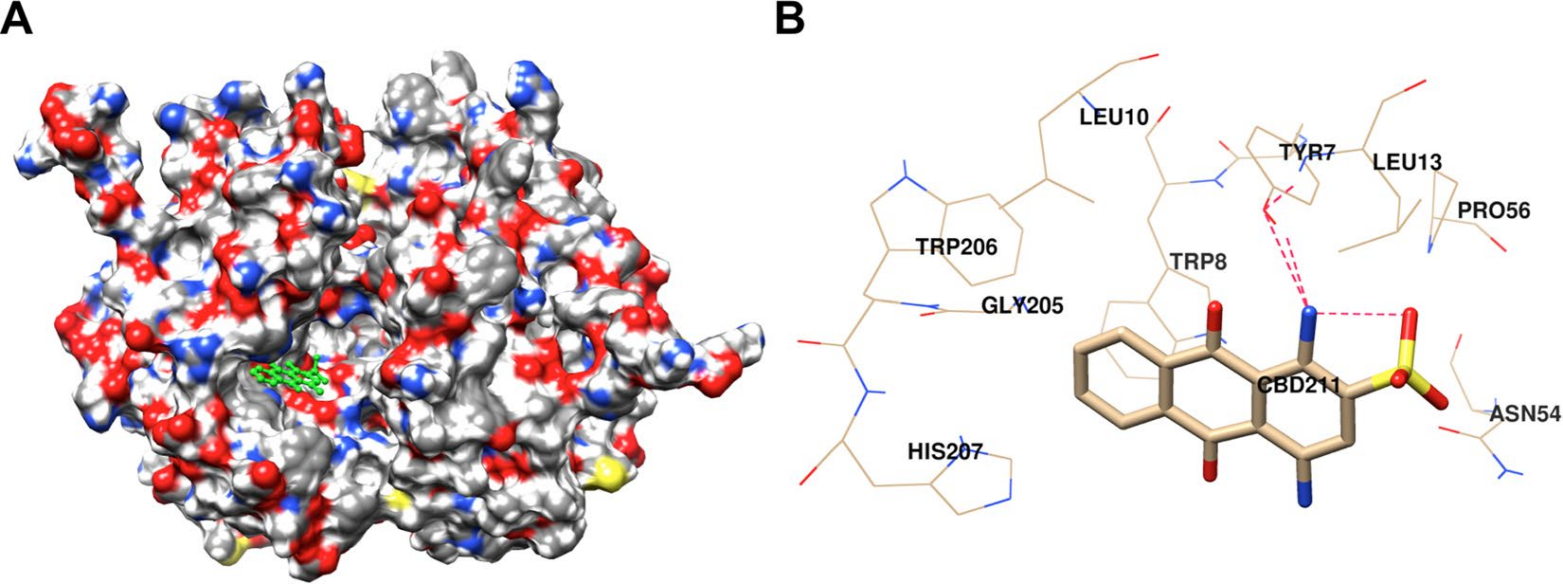
Docking with cibacron blue. (**A**) Surface view of FgGST1 with CB in the predicted cavity. The stick form of CB is highlighted in green color. (**B**) Ligand interaction diagram of the CB with the FgGST1. Red dotted lines represents the hydrogen bonds.

The binding site of CB was predicted by superimposing the modeled FgGST1 with HsGST structure (PDB ID: 20GS) (Fig. 7A). This demonstrated structural similarity in the CB-binding site. Thus, CB was docked in the predicted binding site using Autodock Vina. The complex showed a binding energy of –7.7 Kcal.mol^−1^. The FgGST1-CB complex was stabilized by three hydrogen bonds with Tyr7, Leu13 and eight hydrophobic interactions with Trp8, Leu10, Asn54, Pro56, Gly205, Trp206, and His207 from the chain-A residues (Fig. 7B).

### Structural stability and dynamics of FgGST1 and FgGST1-CB complex

The stability of the predicted FgGST1 model and the binding mode of FgGST1-CB complex were evaluated using 50 ns MDS. The MDS was used for prediction of accurate binding mode. RMSD, RMSF, Rg, hydrogen bonds, PCA, and binding free energy analyses were calculated from the MD trajectories.

**Figure 8:**
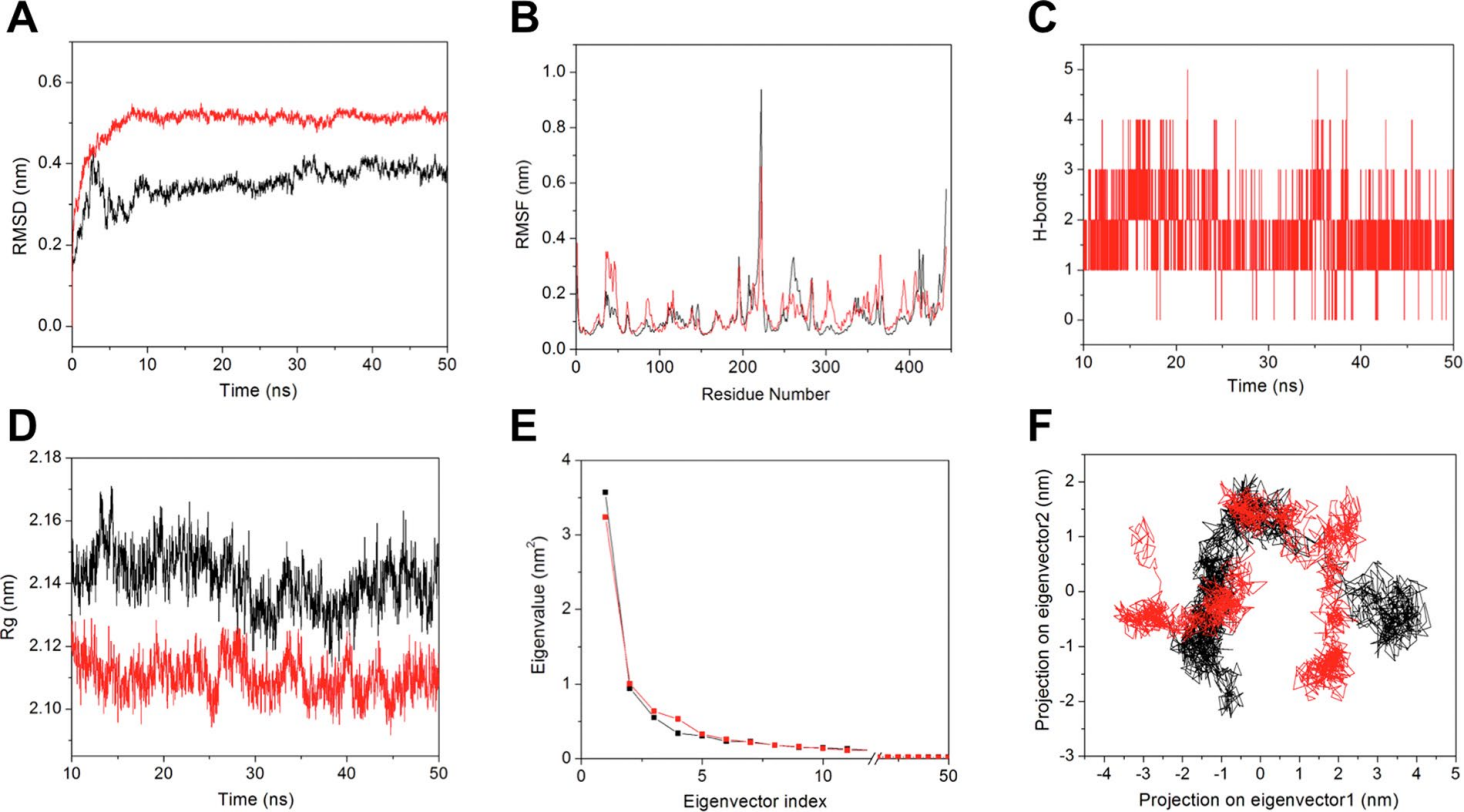
Molecular dynamic simulation. (**A**) RMSD of the backbone Cα atoms for FgGST1 and FgGST1-CB complex relative to the corresponding structure as a function of time. (**B**) RMSF of Cα atoms of FgGST1 and FgGST1-CB complex of last 40 ns MD trajectory. (**C**) Number of hydrogen bonds between FgGST1 and CB for last 40 ns time period. (**D**) Radius of gyration *vs* time for FgGST1 and FgGST1-CB complex. (**E**) First 50 principal components *vs* eigenvectors are shown for FgGST1 and FgGST-CB complex. (**F**) 2D projection plot for first two eigenvectors. FgGST1 and FgGST1-CB complex are shown in black and red color respectively.

The RMSD of FgGST1 and FgGST1-CB initially increased till 10 ns, which means that both the structures dissolved in the solution in the cubic box get relaxed, and the repulsion within the systems is removed during this time. Both systems were well equilibrated after 10 ns and produced stable trajectories for analysis. FgGST1 and FgGST1-CB complexes showed an average RMSD value of 0.34 and 0.50 nm, respectively (Fig. 8A). These values suggest that the modeled FgGST1 structure was more stable as compared to the FgGST1-CB complex.

RMSF values were calculated to compare the flexibility of each amino acid residues in the FgGST1 and FgGST1-CB complex. RMSF describes the residue-wise fluctuation of the whole system. Low and high values of RMSF indicate well-structured regions and loosely organized loop or terminal domains, respectively. In our study, we calculated the average RMSF values for the last 40 ns of MD trajectories. The RMSF peaks of FgGST1 and FgGST1-CB complex was found to be similar (Fig. 8B). The average RMSFs for FgGST1 and FgGST1-CB complex was 0.12 nm and 0.13 nm, respectively. A residual fluctuation within 1–3 Å is reported to be acceptable for small proteins^41,42^.

H-bonds play a significant role in ligand binding and are directly responsible for affinity and specificity in the protein-ligand complex. The number of H-bonds was calculated between CB and FgGST1 during last 40 ns time period to predict the affinity between the complex. The complex forms an average of 1–2 H-bonds during last 40 ns simulation time (Fig. 8C). The percent occupancy of hydrogen bonds was also calculated for last 40 ns of MD trajectory to predict the residues that play an important role during CB stabilization with FgGST1. Several residues like Tyr7 (62.02%), Trp8 (1.25%), Asn54 (0.95%), Ser68 (0.90%), Ser107 (7.30%), and Arg108 (0.80%) seem to play key role in hydrogen bond formation from chain-A. The result revealed that these residues are important for ligand-induced inhibition of FgGST1 activity.

To determine the dynamic stability and compactness of FgGST1 and FgGST1-CB complex, the Rg values were determined after ligand binding. The backbone Rg was calculated for the last 40 ns trajectory and plotted in Fig. 8D. The data showed that the average Rg values for FgGST1 and the FgGST1-CB complex were 2.14 and 2.11 nm, respectively, suggesting that FgGST1 shows slightly higher Rg value compared to the FgGST1-CB complex. The results of Rg value suggest that the complex was marginally more stable in nature than the apo-protein.

PCA was carried out to predict the significant motions in FgGST1 and the FgGST1-CB complex structures. The first few eigenvectors play a key role in the motions of protein. In our study, we calculated concerted motions for the first 50 eigenvectors from the last 40 ns of MD trajectories. The covariance matrix of atomic fluctuations was diagonalized for predicting the eigenvalues. Figure 8E shows the eigenvalues in decreasing order versus the corresponding eigenvector for FgGST1 and FgGST1-CB complex. The first five principal components (PCs) account for 66.74% and 68.37% of motions observed for the last 40 ns trajectories of the FgGST1 and FgGST1-CB complex, respectively. From Fig. 8E, it is evident that FgGST1 showed lesser motions as compared to the FgGST1-CB complex. This suggests that the first few PCs are not the same for FgGST1 andFgGST1-CB complex as the FgGST1 and FgGST1-CB complex showed distinct motions. From the 2D projection plot (Fig. 8F), it was observed that FgGST1-CB complex is more stable as it showed some stable cluster and occupied less phase space as compared to the modeled FgGST1 structure. The result is similar to the predicted PCA result.

The secondary structure analysis was performed for predicting the structural changes after ligand binding for the last 40 ns of MD trajectory. The overall secondary structure result suggested that after ligand binding the flexible structure elements (loop and turns) increased and due to that the rigid structure like α-helix and β-sheets decreased. From residues 110–130, more rigid structures were observed while in the case of ligand bound FgGST1 more flexible structures appeared. From residues 350–370 more α-helices were observed in the case of apo-FgGST while in the case of ligand bound FgGST1 the flexible structure are more. Thus we can conclude that ligand binding induces structural changes. The secondary structure content is shown in Supplementary Figures S7 and S8.

The binding free energy, which is the summation of the non-bonded interaction energies, was calculated for FgGST1-CB complex using the MM-PBSA method. The calculations were performed using the last 10 ns of MD trajectory. The total calculated interaction energies in terms of Van der Waals, electrostatic interactions, polar solvation energy, SASA energy and binding energy were −182.18, −57.91, 159.40, −15.72, and −96.40 kJ.mol^−1^, respectively, for the FgGST1-CB complex (Table 4). Binding free energies confirmed that FgGST1-CB showed good binding affinity.

**Table 4:** Table showing the Van der Waal, electrostatic, polar salvation, SASA and binding energy in kJ mol^−1^ with cibacron blue.

S. No.	Compound	Van der Waals energy	Electrostatic energy	Polar salvation energy	SASA energy	Binding energy
1.	Cibacron blue	−182.18 ± 10.92	57.91 ± 9.21	159.40 ± 12.09	−15.72 ± 0.69	−96.40 ± 11.11

### Changes in molecular properties of FgGST1 associated with GdnHCl-induced unfolding

Unfolding studies on FgGST1 in the presence of increasing GdnHCl concentrations were performed to study the effect of denaturant on the structural properties of FgGST1. The unfolding characteristics of the FgGST1 were studied by monitoring the denaturant-induced changes in the secondary structure and the Trp fluorescence of the protein. Time-dependent changes in structural parameters of FgGST1 showed maximum changes within 4 h of incubation and no further alteration up to the next 12 h.

**Figure 9:**
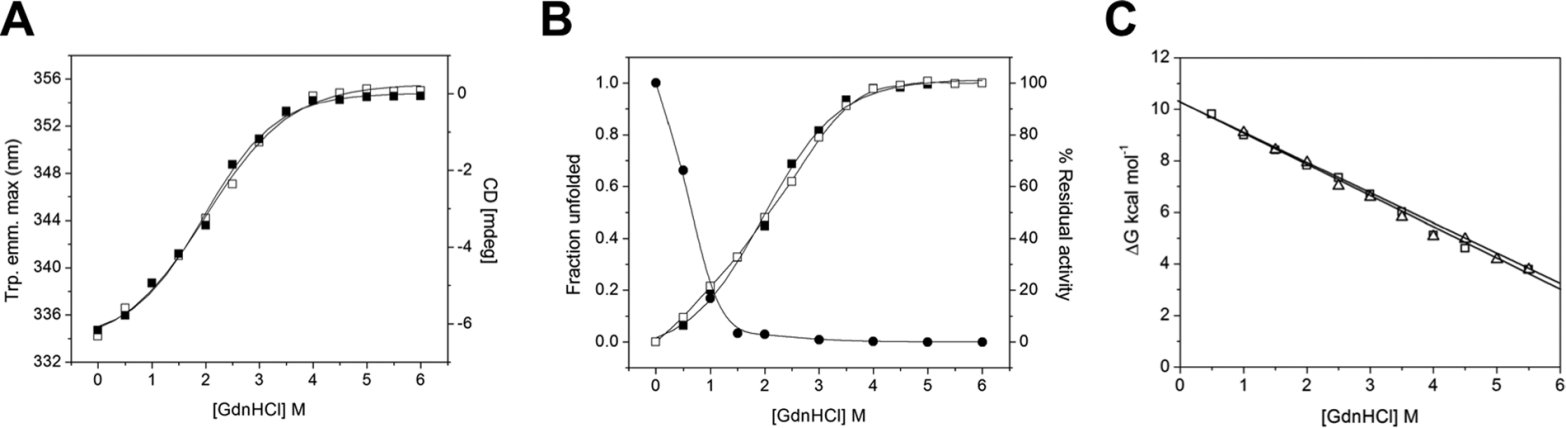
GdnHCl-induced unfolding of FgGST1. (**A**) GdnHCl-induced unfolding as monitored by CD ellipticity at 222 nm (■) and tryptophan fluorescence (□). (**B**) Unfolding of FgGST1 with increasing [GdnHCl] *vs* activity. (**C**) The linear free energy extrapolation curve with respect to [GdnHCl]. The ΔG_D_^H20^ was the intercept on the Y-axis, obtained using the linear extrapolation method.

To study the GdnHCl-induced changes in the secondary structure of FgGST1, far-UV CD studies were carried out. Figure 9A summarizes the effect of increasing GdnHCl concentrations on the CD ellipticity at 222 nm and Trp fluorescence emission maxima. A sigmoidal loss of the CD signal and shift in emission wavelength maxima from 334 nm to 355 nm was observed between 0 and 4 M GdnHCl. At GdnHCl above 4 M, almost complete loss of CD signal at 222 nm and a shift of fluorescence emission maxima to 355 nm were observed. This demonstrated that the enzyme is completely unfolded under these conditions. These observations indicate that GdnHCl induces cooperative unfolding of the protein without stabilization of any intermediate state. C_1/2_ of about 2 M was found to be associated with the GdnHCl-induced unfolding of FgGST1.

The above-mentioned changes in secondary and tertiary structures of protein prompted us to study the changes in the activity of the protein at different denaturant concentrations. The biological function of the protein is considered the most sensitive probe to study the changes in conformation during various treatments. The change in the structure of protein will affect its activity. Figure 9B summarizes the effect of varying concentrations of GdnHCl on the fractional unfolding and activity of FgGST1. A continuous decrease in the activity of FgGST1 from 100% to almost 0% was observed from 0 M to 1.5 M GdnHCl concentration. The activity of the enzyme initiated to lose at very low concentrations of GdnHCl, indicating that the activity is perturbed with a slight change in the structure. Almost 100% of the enzyme activity was lost upon only 30% loss in the protein structure. The enzymatic inactivation with GdnHCl precedes major conformational and structural changes. The aromatic nucleophilic substitution reaction of GST activity obeys an addition–elimination mechanism. The nucleophilicity of the thiolate anion of GSH is increased by the Tyr9 of the G-site, where the -SH group of the Cys moiety of GSH is stabilized. In the presence of GdnHCl, the nucleophilicity of the GS^–^ anion is reduced and the activity of the enzyme is decreased.

We further determined the conformational stability of FgGST1 assuming the two-state model of unfolding. The GdnHCl-induced denaturation curves were used to determine the free energy of stabilization in the absence of denaturants ΔG_D_^H2O^ by linear extrapolation of the ΔG_D_ values to zero denaturant concentration (Fig. 9C). From these measurements, the ΔG_D_^H20^ value was calculated to be around 10 kcal.mol^−1^. The estimates of free energy of stabilization based on fluorescence and CD spectroscopy are in excellent agreement with each other.

## Materials and Methods

The molecular biology kits and Ni-NTA agarose were purchased from Qiagen, CA, USA. The dNTPs and enzymes were purchased from New England Biolabs, MA, USA. All other reagents and chemicals were of the highest purity available and were purchased either from Sigma- Aldrich Chemical Company, St. Louis, MO, USA or Sisco Research Laboratories, Mumbai, India. Bacterial culture media were purchased from Himedia Laboratories, Mumbai, India.

### Phylogenetic analysis

Phylogenetic analysis of FgGST1 was carried out using Jalview (http://www.jalview.org/). The FgGST1 sequence was submitted to BLAST search to predict the homologs. Out of the predicted homologs, we chose only the homologs of mu-class GST that were aligned by ClustalW to predict the evolutionary relationship between them. After alignment, the phylogenetic tree was constructed by using ESPript^43^. A total of 15 mu-class GST sequences were retrieved from the NCBI database that belonged to the Phylum Platyhelminthes and aligned using ClustalW algorithm. *H*. *sapiens* mu-class GST was also taken for comparison. These sequences were used for predicting the evolutionary relationship of FgGST1.

### Collection and identification of parasites

The adult liver flukes were collected from the liver of naturally infected cattle and washed extensively with chilled PBS (pH 7.5) at the local slaughterhouse at Bada Bazaar, Shillong, Meghalaya. The flukes were identified as *F*. *gigantica* using morphological properties.

### Isolation of total RNA and cDNA synthesis

The liver flukes were crushed in a pestle and mortar with liquid nitrogen, and the total RNA was isolated using the RNeasy mini kit (Qiagen, USA) as provided by the manufacturer instructions. The first strand cDNA was synthesized using Quantitect Reverse transcriptase kit (Qiagen, USA) as per the manufacturer instructions.

### Cloning, overexpression and purification of FgGST1

The cDNA was used as a template for polymerase chain reaction (PCR). The *gst* gene of 0.66 kb encoding for functional GST protein was amplified using gene-specific primers (forward-5′-GGATCCATGCCTGCAACG-3′ and reverse-5′-AAGCTTTCACTTCTTTTCATGGC-3′). The PCR conditions used included 98 °C for 30 s followed by 35 cycles (98 °C for 10 s, 53 °C for 15 s, and 72 °C for 5 s), and a final elongation at 72 °C for 3 min. The amplified fragments were cloned into the pSK+ vector, sequenced and further sub-cloned in pET23a (+) vector at BamHI and HindIII sites. The resultant constructs were transformed into *E*. *coli* BL21(DE3) cells for expression.

Recombinant FgGST1 was overexpressed in *E*. *coli* BL21(DE3) cells and purified as follows. A single colony from transformed plates was inoculated in 5 mL Luria Bertini (LB) broth containing 100 μg/mL ampicillin. The cells were grown for 12 h at 37 °C with continuous shaking at 160 rpm. Subsequently, two 500 mL LB broth tubes containing the above mentioned antibiotics were inoculated with 1% (v/v) of 4–5 h grown culture and incubated at 37 °C with shaking. Cultures were grown until the OD_600_ reached a value of 0.5–0.6; at this stage, the culture was induced with 1 mM isopropyl β -D-1-thiogalactopyranoside (IPTG). The other un-induced culture was used as a control. After 16 h of induction at 20 °C, both the cultures were pelleted by centrifugation at 8000 rpm for 10 min at 4 °C. The pellet was then resuspended in lysis buffer that contained 50 mM phosphate (pH 7.2), 150 mM NaCl, 10% glycerol and a cocktail of protease inhibitors. The dissolved cells were lysed by sonication, and the lysate was centrifuged at 13,000 rpm for 30 min at 4 °C and the supernatant was collected. All further steps were performed under cold conditions. GST affinity matrix was equilibrated with equilibration buffer (50 mM phosphate pH 7.2, 150 mM NaCl and 10% glycerol). The supernatant was poured on the affinity column and was allowed to bind slowly. Non-specifically bound, contaminating proteins were removed by washing with equilibration buffer. The recombinant protein was eluted with 10 mL of elution buffer (equilibration buffer containing 50 mM GSH). The protein was dialyzed against 20 mM phosphate buffer, pH 7.5 containing 150 mM NaCl with or without 2 mM GSH. Protein concentration was determined by Bradford method using bovine serum albumin as a standard. The eluted protein was tested for purity by SDS-PAGE.

### Size exclusion chromatography

Gel filtration experiment was carried out on a Superdex^TM^ 200 10/300 GL column (manufacturer’s exclusion limit 600 kDa for proteins) on an ÄKTA-FPLC (GE HealthCare Biosciences). The column was equilibrated and run with 20 mM phosphate buffer (pH 7.5), containing 150 mM NaCl with a flow rate of 0.3 mL/min with detection at 280 nm.

### Biochemical assays

FgGST1 activity using GSH and CDNB as substrates was determined spectrophotometrically at 340 nm on the basis of the extinction coefficient for the product S-(2,4-dinitrophenyl) glutathione (ε_340_ = 9.6 mM^−1^cm^−1^). The assay mixture (1 mL) containing 32 nM FgGST1 enzyme and 3 mM GSH in 20 mM phosphate buffer, pH 7.5 and 150 mM NaCl was incubated at 30 °C for 10 min. The reaction was started on addition CDNB. One unit of GST activity was defined as the conjugation of 1 μmol of CDNB with GSH per minute at 25 °C. The pH optimum was determined for CDNB conjugation activity using citrate/glycine/hepes (CGH) buffer of various pH values. Purified FgGST1 was incubated at 30 °C for 30 min in CGH buffer of pH values ranging from 2 to 11. Conjugation activity was determined as described above. For temperature dependent activity, the protein was incubated from 20 °C to 80 °C for 10 min and then activity was taken at the same temperature.

Several substrates were screened for GST activity on the basis of extinction coefficient of the product formed. All the activities were performed as described earlier^44–46^. FgGST1 activity with the carcinogenic substrate 4-nitro quinolone-1-oxide was measured at 350 nm as described in previous reports^47^. All experiments were repeated thrice and ±SD was taken.

### Determination of enzyme kinetics

All the enzymatic reactions were carried in a quartz cuvette of path-length 1 cm, with a total volume of 1 mL. The data were recorded using a Varian Cary 50 Bio UV-Visible spectrophotometer. The steady-state kinetic parameters (*K*m and *V*max) were determined under variable concentrations of substrates at a fixed concentration of enzyme. The values were estimated by fitting the curve through non-linear regression by plotting Michaelis-Menten graph. Kinetic calculations were performed using the GraphPad Prism software. Three replications were conducted, and background data were subtracted for all the experiments. The error bars represent the mean of triplicate samples.

### Inhibition studies

To study the effect of inhibitor on the activity of FgGST1, a commercially available inhibitor CB was used. The enzyme was incubated in 20 mM phosphate buffer (pH 7.5) containing 150 mM NaCl, 3 mM GSH for 10 min at 25 °C with inhibitor (1 to 50 μM). The reaction was started with addition of 1 mM CDNB^48^ and monitored spectrophotometrically at 340 nm.

### Homology modeling, structure validation, and molecular docking

The secondary structure content was predicted by PSI-PRED (http://bioinf.cs.ucl.ac.uk/psipred/) and SOPMA server (https://npsa-prabi.ibcp.fr/NPSA/npsa_sopma.html). The FgGST1 sequence was submitted to PDB BLAST to predict the closely related homologs. The structure of the *F*. *hepatica* GST (PDB ID: 2FHE) was selected as template for homology modeling^40^. FgGST1 was aligned with FhGST sequence using ESpript3.0^43^ and was modeled on the basis of the crystallographic information of FhGST using Modeller9.16^49^. The predicted model was validated by structure alignment using ProSA^50^, PDBsum^51^, and verify3D^52^ servers as earlier^53–55^. ProSA was used to calculate Z-score and energy value. PDBsum was used for constructing the Ramachandran plot for visualization of backbone dihedral angles. Verify3D was used to determine the compatibility of the 3D model of FgGST1 with its own amino acid sequence by assigning a structural class based on its location and environment and comparing the results to known structures. Root mean square deviation (RMSD) was calculated between the predicted FgGST1 and FhGST structure by Chimera1.10.2^56^. FgGST1 and ligands (GSH and cibacron blue) were prepared by using MGL tools^57^. All the polar hydrogens were removed from ligands and Gasteiger type charge was assigned. After that the receptor file was prepared by adding hydrogens and Kollman charges. Then all files were converted to pdbqt format for docking. The grid was set on the basis of conserved residues in G-site and H-site. Docking was performed with default parameters of AutodockVina^58^.

### Molecular dynamics simulation (MDS)

GROMACS 4.6.5^59^ was used to perform MDS in an *in house* supercomputer as earlier^60–64^. Two systems were created and used for 50 ns MDS studies, one for predicting the stable structure of the apo-FgGST1 and others for FgGST1-CB bound complex. Both the systems were solvated using simple point charge model in a cubic box. Ligand topology was generated by using ProDRG server^65^. Protein topologies were generated by using GROMOS 9653a6 force field^66^. Two Na^+^ ions were added for neutralization of the systems. Steepest energy minimization was performed for both the systems to give the maximum force below 1000 kJ mol nm^−1^ for removing the steric clashes. Long range electrostatic interactions were calculated by Particle Mesh Ewald (PME) method. For the computation of Lennard-Jones and Coulomb interactions, 1.0 nm radius cut-off was used. The LINCS algorithm^67^ was used to constrain the hydrogen bond lengths. The time step was maintained at 2 fs for the MDS. For predicting the short-range non-bonded interactions, 10 Å cut-off distance was used. 1.6 Å Fourier grid spacing was used for the PME method for long-range electrostatics. All bonds including hydrogen bonds were fixed by Shake algorithm^68^. Both the systems were equilibrated after energy minimization. Then position restraint simulation of 1 ns was carried out under NVT and NPT conditions. Finally, both systems were submitted for 50 ns MDS. 2 fs interval was given for saving the coordinates. Then the root mean square deviation (RMSD), root mean square fluctuation (RMSF), Radius of gyration (Rg), hydrogen bonds and principal component analysis (PCA) were calculated by g_rms, g_rmsf, g_gyrate, g_hbond, g_cover and g_anaeig tools as describe previously^54,62,69,70^. Binding free energy was calculated by using g_mmpbsa tool^71^. The trajectories were analyzed by visual molecular dynamics^72^ and Chimera 1.10.2^56^. Origin 6.0 was used for generating and visualizing the plots.

### Equilibrium unfolding experiments

A stock solution of 8 M GdnHCl was prepared in 20 mM phosphate buffer (pH 7.5) containing 150 mM NaCl. All samples were incubated for 4 h to achieve denaturation equilibrium before taking readings.

### Fluorescence and circular dichroism (CD) spectroscopy

Trp fluorescence spectra were recorded with a Perkin Elmer LS 55fluorescence spectrometer in a 5 mm path length quartz cell at 25 °C. Excitation wavelength of 280 nm was used and the spectra were recorded between 300 and 500 nm. The protein concentration of 1 µM was used for the studies. CD measurements were made on JASCO 1500 spectropolarimeter calibrated with ammonium (+)-10-camphorsulfonate with a 1 mm path length cell at 25 °C. Spectra were collected at a scan speed of 50 nm/min, a response time of 1 s and a bandwidth of 2 nm. 2.5 μM protein was used for the studies in the above-mentioned buffer. The spectra were averaged over five scans to eliminate signal noise. The values obtained were normalized by subtracting the baseline recorded for the buffer under similar conditions.

### Calculation of free energy of stabilization

Assuming a two-state model of denaturation of the protein, the spectroscopic data was converted into the free energy of unfolding for each data point (ΔG_D_). The ΔG_D_ values were then plotted against GdnHCl concentration to determine the free energy of stabilization in the absence of denaturants (ΔG_D_^H20^) according to the linear extrapolation method^73^ as described earlier^74–76^.

## Electronic supplementary material

Supplementary Figures
