# Supplementary material for: Comprehensive analysis of the catalytic and structural properties of a mu-class glutathione s-transferase from Fasciola gigantica
Source: Sci Rep. 2017 Dec 13;7:17547. doi: 10.1038/s41598-017-17678-3 (PMC5727538; doi:10.1038/s41598-017-17678-3)
Supplement: Supplementary file 1 — Supplementary Figures [file 41598_2017_17678_MOESM1_ESM.docx]

**Comprehensive analysis of the catalytic and structural properties of a mu-class glutathione s-transferase from *Fasciola gigantica***

Jupitara Kalita^1^, Rohit Shukla^1^, Harish Shukla^1^, Kundlik Gadhave^2^, Rajanish Giri^2^, and Timir Tripathi^1^*

^1^Molecular and Structural Biophysics Laboratory, Department of Biochemistry, North-Eastern Hill University, Shillong 793022, India

^2^School of Basic Sciences, Indian Institute of Technology Mandi, Kamand, Himachal Pradesh 175005, India

*Running title: Characterization of a mu-class GST from F. gigantica*

*To whom correspondence should be addressed: Dr. Timir Tripathi, Department of Biochemistry, North-Eastern Hill University, Shillong- 793022, India. Email: timir.tripathi@gmail.com, ttripathi@nehu.ac.in. Tel: +91-364-2722141; Fax: +91-364-2550108.

**Supplementary Figure S1.** Phylogenetic tree of amino acid sequences of mu class GSTs. The phylogenetic tree was constructed by Jalview software using BLOSUM62. Each entry includes the species name and accession number.

**
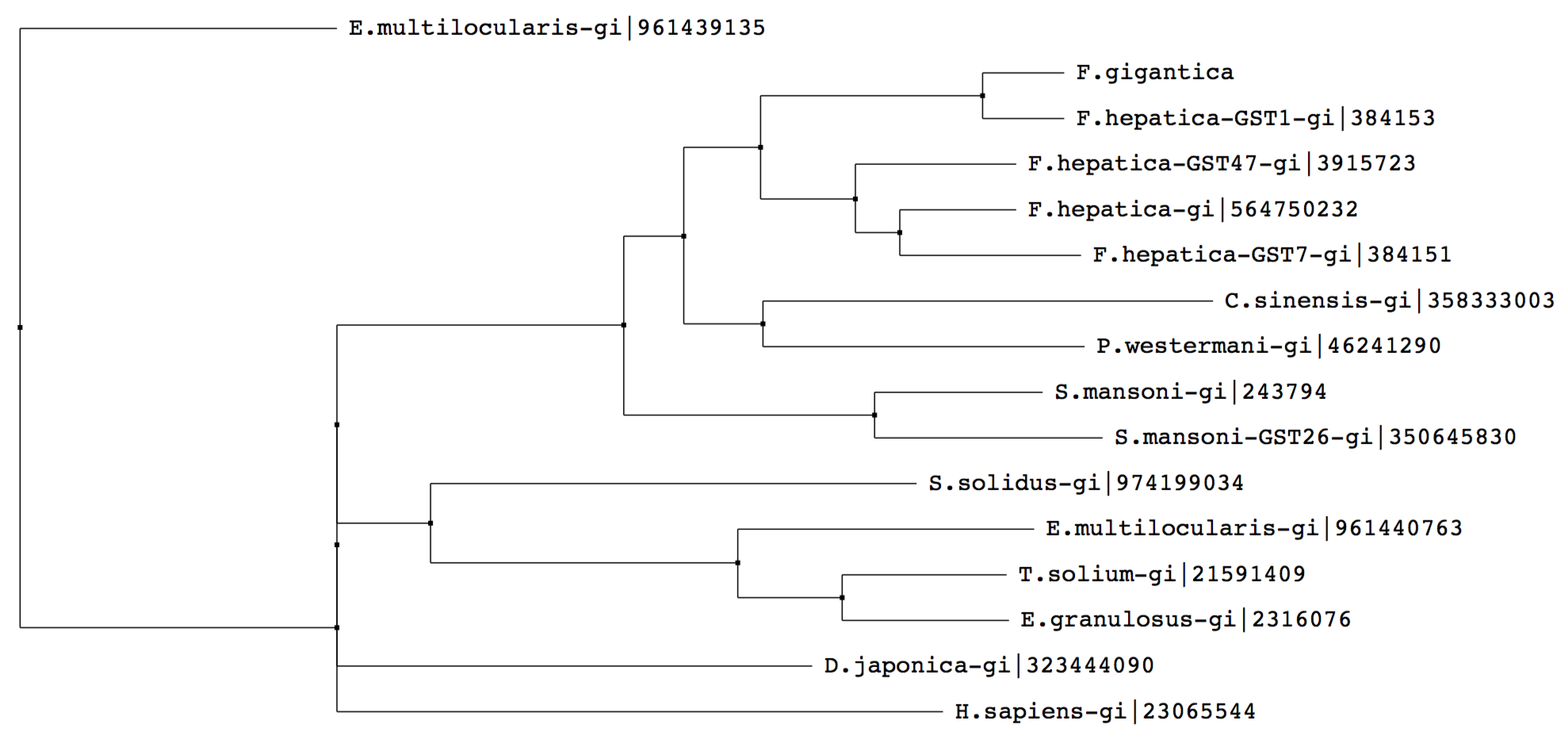
**

**Supplementary Figure S2.** Secondary structure of FgGST1 was predicted by PSIPRED server. Pink cylinders and yellow arrows indicate helices and β-sheets respectively.

**
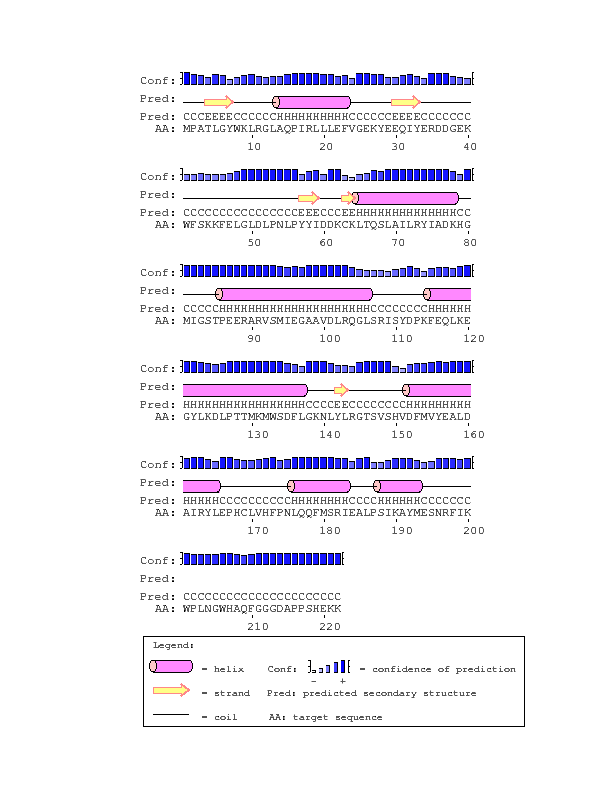
**

**Supplementary Figure S3.** FgGST1 and FhGST (PDB ID: 2FHE) sequence were taken for pairwise sequence alignment. The sequence of FgGST1 and FhGST displayed 91.9% identity in pairwise sequence alignment. Red and yellow boxes represent the identical and similar amino acid residues respectively.

**
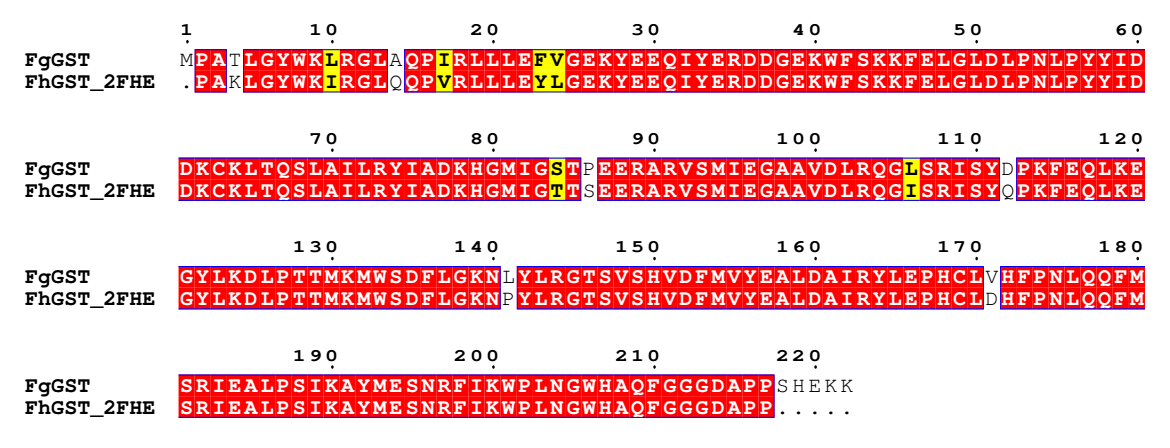
**

**Supplementary Figure S4.** Ramachandran plot for the homology modelled FgGST1 structure.


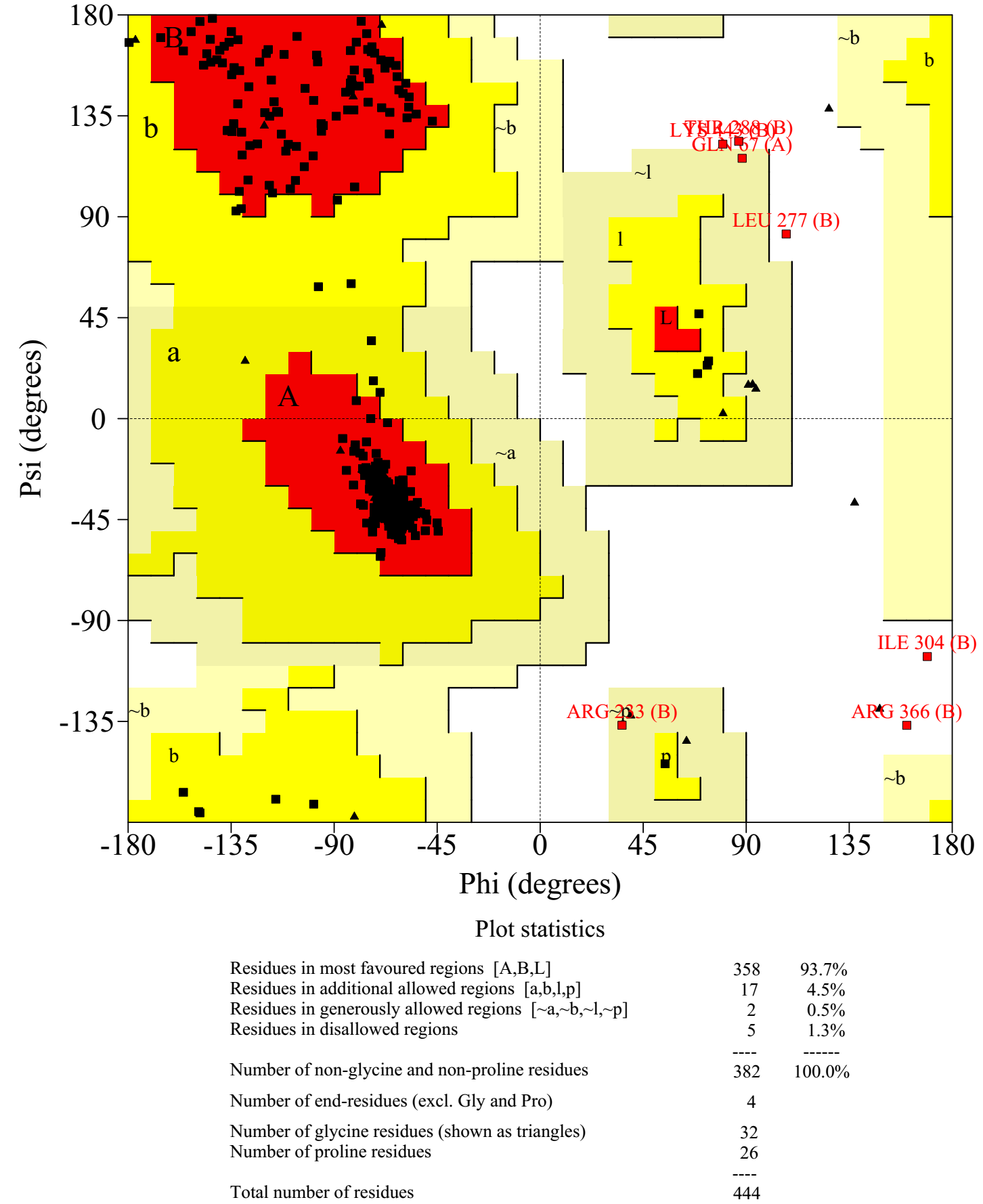


**Supplementary Figure S5.** ProSA-web z-score plot for predicted 3D structure of FgGST1 showed -7.83 Z-score. The Z-score of FgGST1 was represented by a black spot. Z-Score plot consists Z-scores of all experimentally protein chains in PDB defined by NMR spectroscopy (dark blue) and X-ray crystallography (light blue).


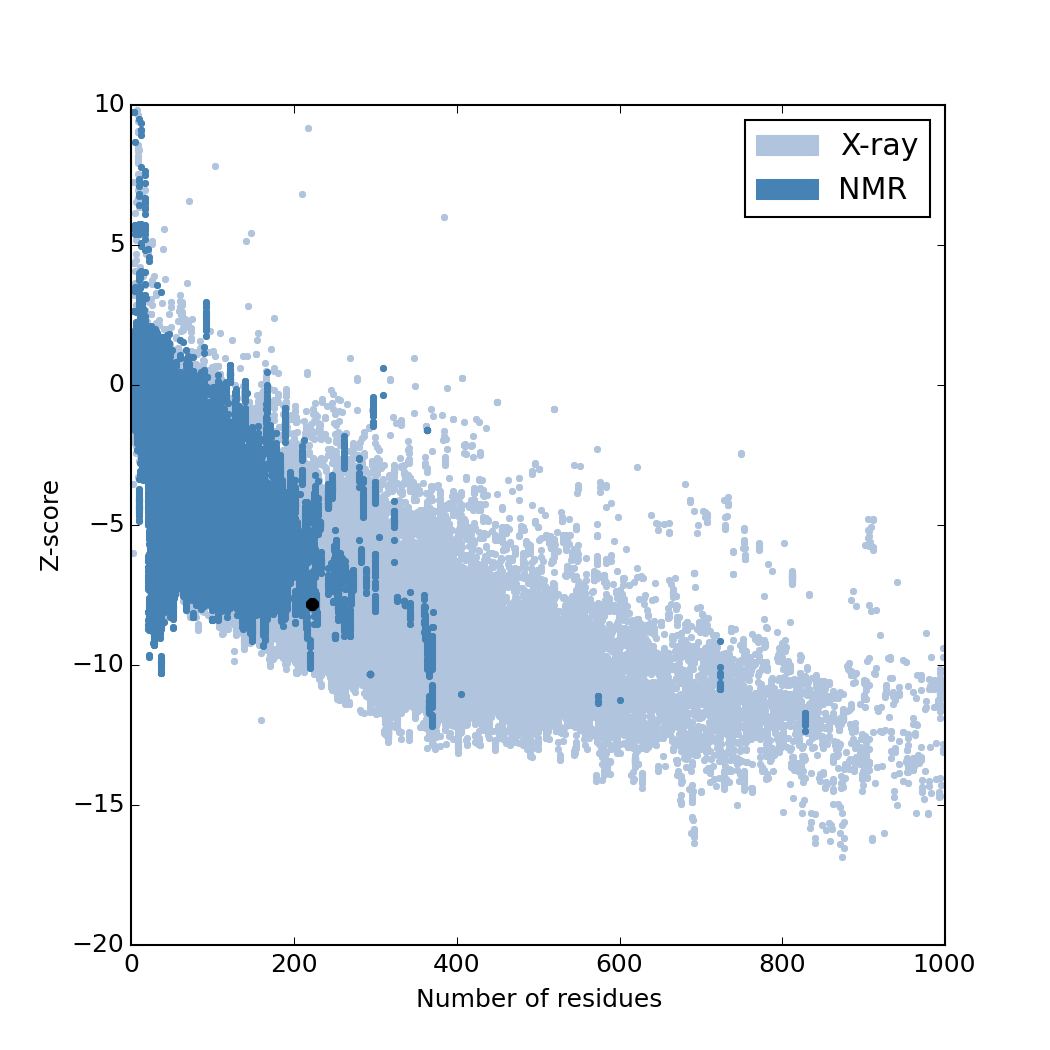


**Supplementary Figure S6.** Energy plot for all residues was predicted by ProSA-web server. All residues lie in the negative window and represent the lowest energy structure. In general, positive values correspond to problematic or erroneous parts of the input structure.

**
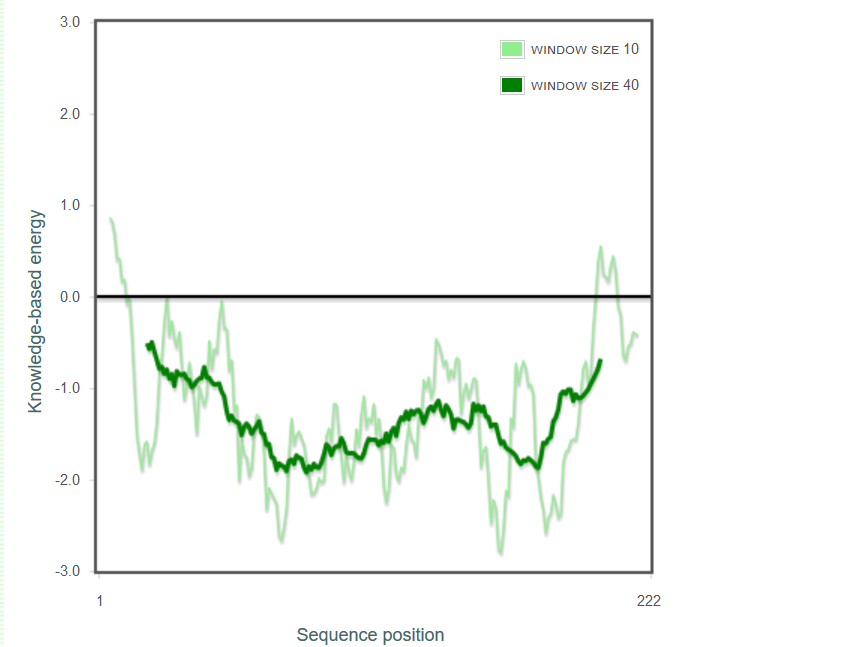
**

**Supplementary Figure S7.** Time evolutionary secondary structural elements for apo-FgGST1 for last 40 ns trajectory.

**
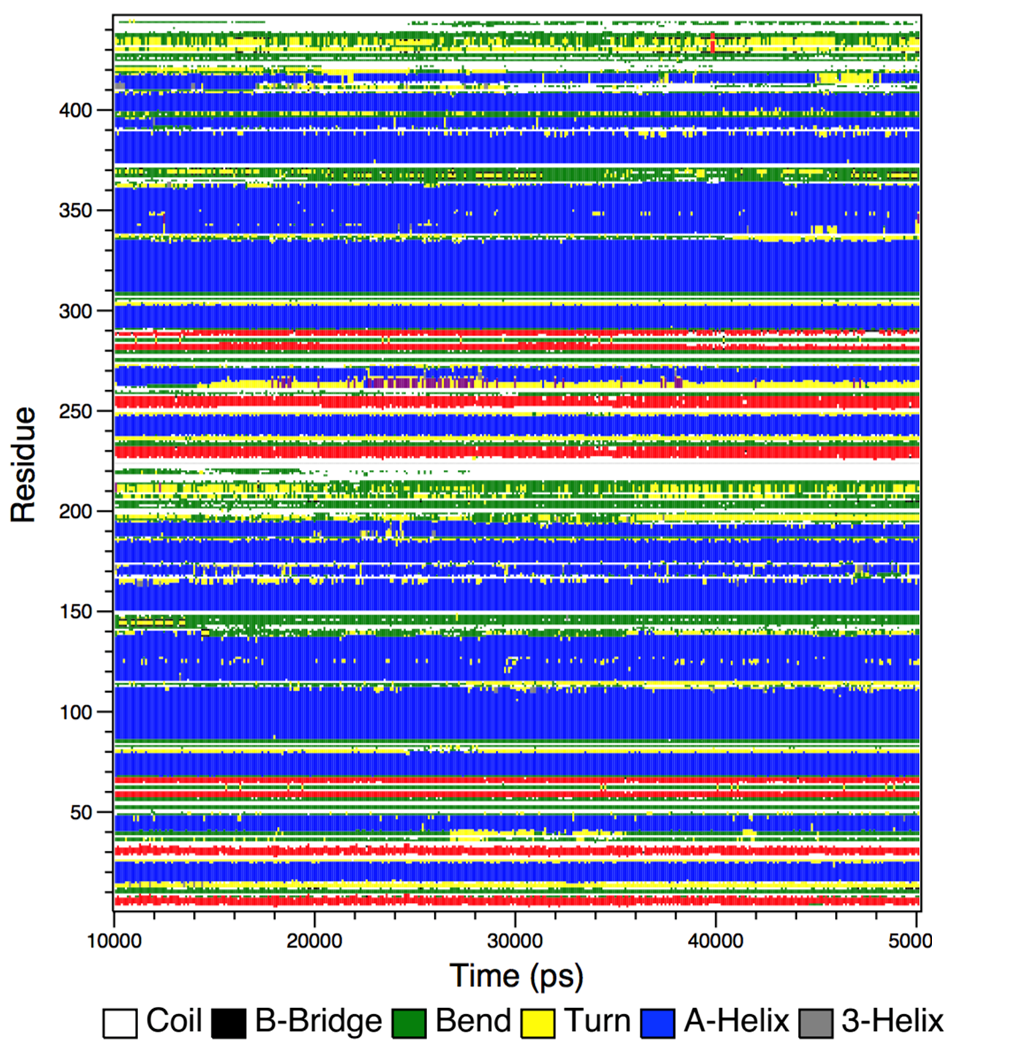
**

**Supplementary Figure S8.** Time evolutionary secondary structural elements for FgGST1-ligand for last 40 ns trajectory.

**
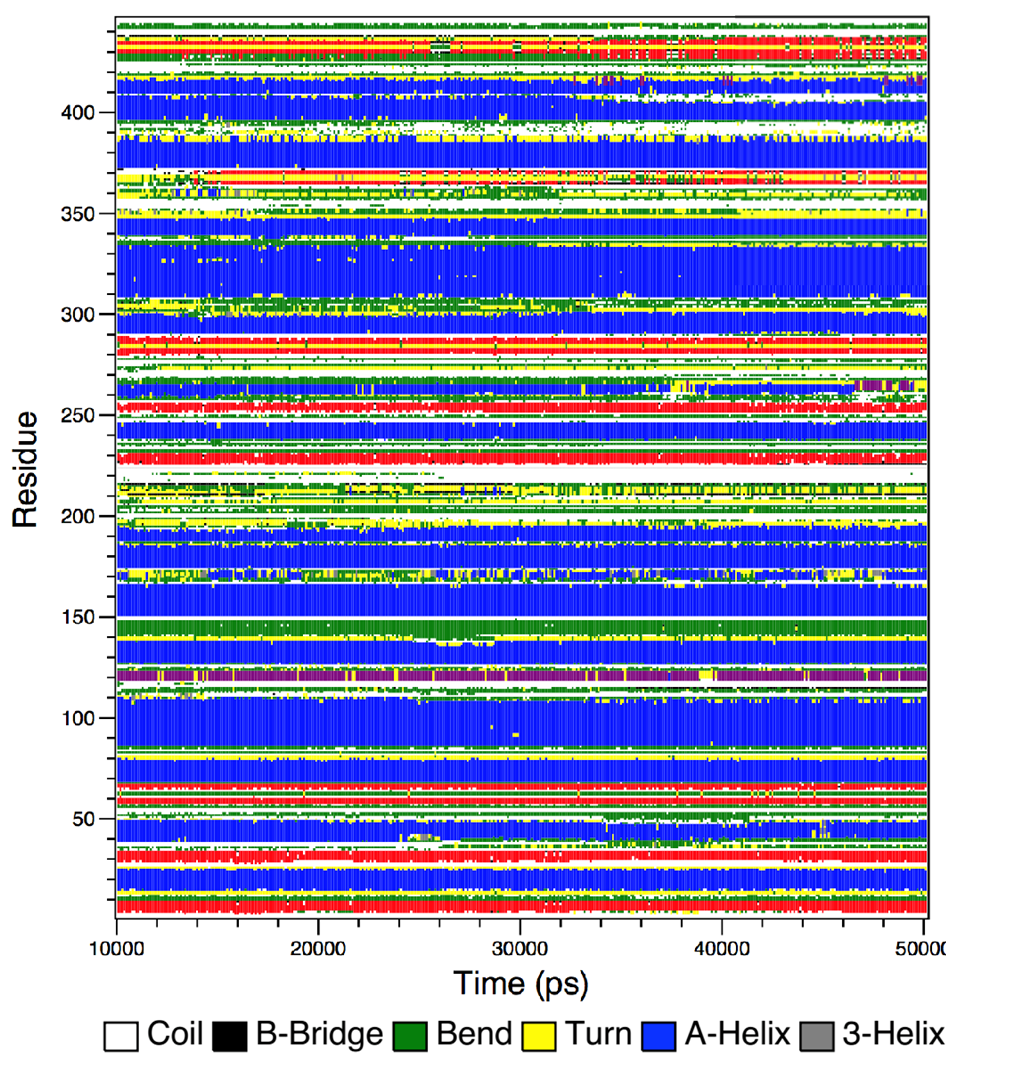
**
